# Low-loss MIMO antenna wireless communication system for 5G cardiac pacemakers

**DOI:** 10.1038/s41598-023-36209-x

**Published:** 2023-06-12

**Authors:** Deepti Sharma, Binod Kumar Kanaujia, Sachin Kumar, Karumudi Rambabu, Ladislau Matekovits

**Affiliations:** 1 School of Computational and Integrative Sciences, Jawaharlal Nehru University, New Delhi, India; 2 Dr. B. R. Ambedkar National Institute of Technology, Jalandhar, Punjab India; 3 Department of Electronics and Communication Engineering, SRM Institute of Science and Technology, Kattankulathur, Chennai, Tamil Nadu India; 4 Department of Electrical and Computer Engineering, University of Alberta, Edmonton, AB Canada; 5 Department of Electronics and Telecommunications, Politecnico di Torino, Turin, Italy; 6 Faculty of Electronics and Telecommunications, Politehnica University Timişoara, 300006 Timişoara, Romania; 7 Istituto di Elettronica e di Ingegneria dell’Informazione e delle Telecomunicazioni, National Research Council of Italy, Turin, Italy

**Keywords:** Electrical and electronic engineering, Biomedical engineering

## Abstract

Cardiovascular diseases (CVDs) are one of the leading causes of death globally. The Internet of things (IoT) enabled with industrial, scientific, and medical (ISM) bands (2.45 and 5.8 GHz) facilitates pacemakers to remotely share heart health data to medical professionals. For the first time, communication between a compact dual-band two-port multiple-input-multiple-output (MIMO) antenna (integrated inside the leadless pacemaker) and an outside-body dual-band two-port MIMO antenna in the ISM 2.45 and 5.8 GHz frequency bands is demonstrated in this work. The proposed communication system offers an attractive solution for cardiac pacemakers as it can operate on a 5G IoT platform while also being compatible with existing 4G standards. The experimental verification of the proposed MIMO antenna low-loss communication capability is also presented by comparing it to the existing single–input–single–output communication between the leadless pacemaker and outside body monitoring device.

## Introduction

**Figure 1:**
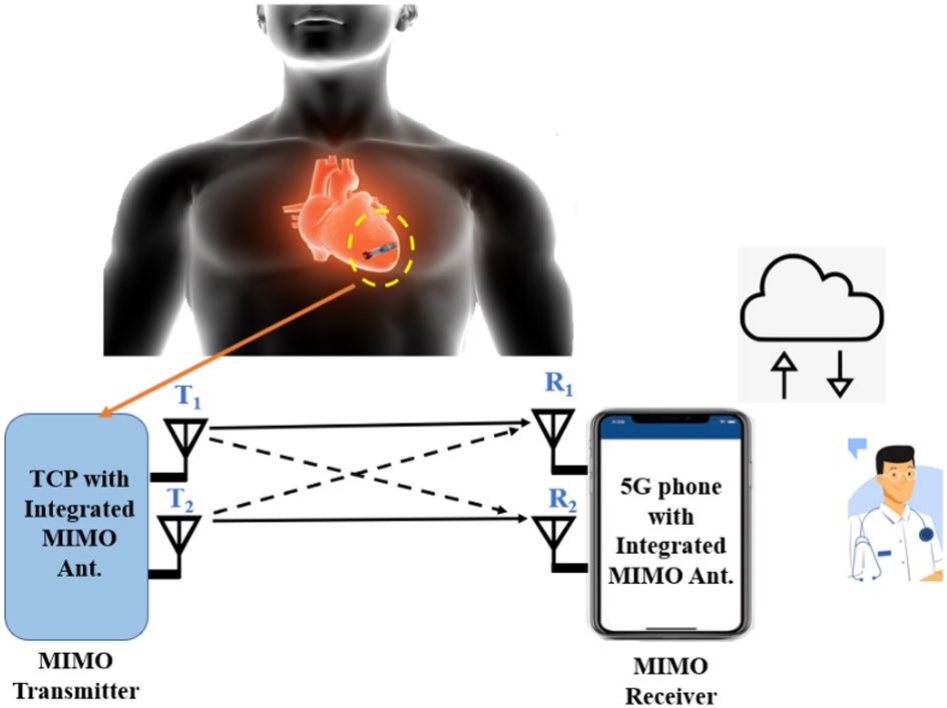
Schematic of MIMO antenna communication between leadless pacemaker and smart phone/external monitoring device in 5G environment (human body and doctor’s figure is adapted from^24,25^).

Low-cost health-care facilities are required to promote healthy ageing and reduce premature deaths from cardiovascular diseases (CVDs)^1^. In this direction, Medtronic® introduced the world's first remote cardiac monitoring system in 2002, which has been used by two million patients worldwide. Remote monitoring not only helps to limit in-person contact with other patients, healthcare workers, and physicians, but it also reduces exposure to various viruses, including COVID 19^2^. Several studies using a patch antenna for a leadless pacemaker have been published in the past^3–6^. Leadless pacemaker antenna designs are classified into two types: conformal^7,8^ and flat^3–6^. The flat antennas must be placed on the PCB holder or on top of the telemetry module inside the pacemaker capsule, while the conformal antennas must be wrapped around the curvature of the pacemaker capsule. The reported antennas were operated in the medical implant communication system (MICS)/400 MHz band and the industrial, scientific, and medical (ISM)/2.45 GHz band^3–6^. Recently, in^9^ and^10^, two implantable antennas were proposed operating in single ISM 2.45 GHz band, and a multi-band antenna was proposed in^11^. However, due to the single antenna element, such antenna systems will not be suitable for next generation pacemakers. A leadless cardiac pacemaker (LCP) outfitted with a multiple-input-multiple-output (MIMO) antenna, as shown in Fig. 1 will be a potential solution for real-time monitoring of heart health via high-speed (5G) data transmission in a lossy biological environment. Furthermore, if such pacemakers are equipped with multi-band implantable antennas, we can achieve high data rate telemetry with minimal losses^12^ while also having power telemetry can be achieved. When compared to a single antenna system, MIMO communication systems offer high capacity and low-loss transmission/reception without requiring additional power^13^.

In the literature, there are few works reported on implantable MIMO antennas^14,15^. Despite the fact that antennas in^14^ and^15^ were proposed for implantable applications, the authors did not investigate specific absorption rate (SAR), and the MIMO antenna reported in^14^ had poor isolation (12 dB). In^16^, a two-port single band (ISM 2.45 GHz) MIMO antenna with two different antenna elements was presented. In^17^, an implantable antenna with a partial and slotted ground plane was proposed, which operated in a single band (ISM 2.45 GHz) and had inter-element isolation of 16 dB. In^18^, a two-port MIMO antenna with isolation of 26 dB was reported. In^19^, a four-port head implantable MIMO antenna with high port isolation and sufficient gain was proposed, but the antenna size was quite large. In^20^ and^21^, implantable MIMO antennas were proposed, both of which operated in single ISM 2.45 GHz frequency band with slot loaded ground planes. In^22^ and^23^, two implantable MIMO antennas were proposed, where the MIMO antenna in^22^ had slotted ground with two shorting pins, whereas the MIMO antenna in^23^ had a complete ground plane. In the above-mentioned implantable MIMO antennas, defects or slots on the ground surface have a significant impact on the performance of the antenna. It is well understood that an antenna with a complete ground plane ensures radiation in the broadside direction with the required high front-to-back ratio, i.e., outwards from the body towards the external monitoring device.

To date, there has been no article in the open literature on a compact multi-band MIMO antenna system for leadless transcatheter pacemaker (TCP). In this paper, an ultra-compact two-port MIMO antenna system with dimensions of 3.4 mm × 6.6 mm × 0.254 mm is presented, which can be easily integrated with commercial pacemakers (Medtronic® Micra TCP and Nanostim® LCP).

The proposed antenna operates in dual-frequency bands (2.45 GHz and 5.8 GHz), has a flat profile, and offers satisfactory gain in the reported bands. The proposed MIMO antenna has the smallest footprint as compared to the previously reported implantable MIMO antennas. Here, higher ISM frequency bands that are compatible with Wi-Fi and Bluetooth technologies are used in the IoT environment. In order to ensure patient safety due to the long-term use of the pacemaker, loop antenna is chosen, which confines current to the radiator and results in low radiation effects due to less interaction with biological tissues^27^.

The proposed implantable MIMO antenna is simulated in the canonical model of the heart muscle after being placed at the top of the telemetry module inside the TCP system, as per^28^. Furthermore, a communication system is simulated in the Ansys HFSS® software to validate far-field communication performance of the proposed MIMO antenna. For this purpose, the MIMO TCP system is implanted in the multi-layer canonical heart phantom, and the external MIMO antenna is placed at different distances in the far-field region. The proposed antenna prototype is fabricated and packed inside a ceramic alumina capsule (dimensions similar to the Medtronic's Micra TCP) along with dummy electronics (with real-time dielectric properties) and a battery, and experimentally validated in a heart muscle gel (ballistic gel) phantom. Also, the communication is configured for MIMO and single port antenna, and the results are compared to illustrate the advantages of MIMO over single–input–single–output (SISO) communication.

## Methodology

### Design of an implantable MIMO antenna inside TCP

**Figure 2:**
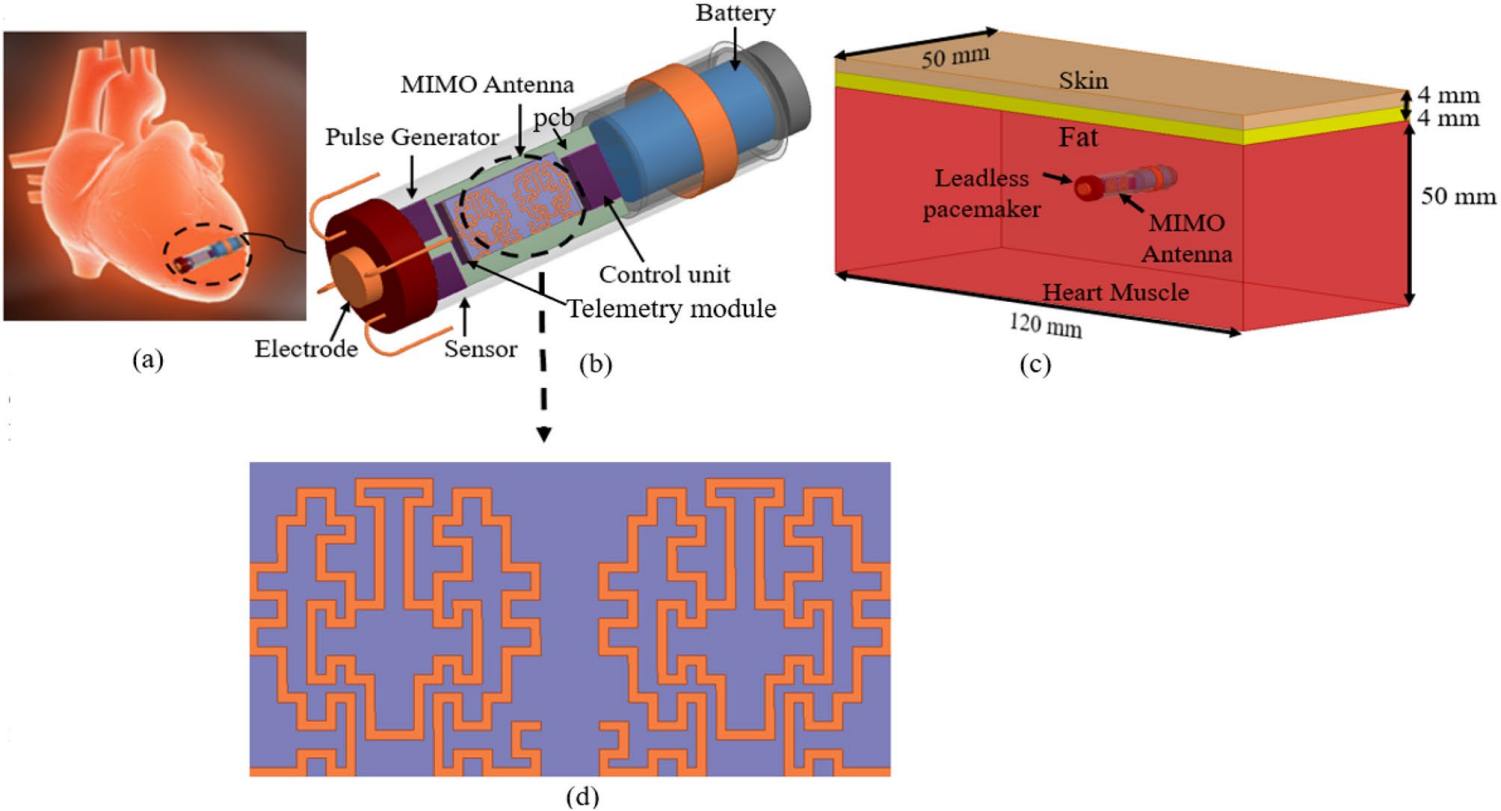
Conceptual/simulated environment of the MIMO antenna integrated within the leadless pacemaker inside the heart model (**a**) Demonstration of a leadless pacemaker implanted into the heart (heart image is adapted from^26^). (**b**) Device architecture of the leadless pacemaker. (**c**) Leadless pacemaker implanted in the multi-layer canonical model of the human heart. (**d**) Two-port MIMO antenna with a full ground plane with dimensions (*W* = 3.4 mm, *L* = 6.6 mm, *a*_1_ = *b*_11_ = 0.6 mm, *a*_2_ = 0.4 mm, *a*_3_ = *a*_5_ = *a*_6_ = *a*_*7*_ = *b*_9_ = 0.3 mm, *a*_4_ = *a*_9_ = 0.2 mm, *a*_8_ = 0.8 mm, *b*_8_ = 1.0 mm, *b*_10_ = 0.7 mm, *t* = 0.1 mm).

To implement the TCP system implanted inside the human heart (as shown in Fig. 2a,b), a three-layer (heart muscle, fat, and skin) canonical model is designed in the finite element method (FEM)-based ANSYS HFSS tool. The ISM 2.45 GHz and 5.8 GHz frequency bands are chosen for MIMO antenna operation. At 2.4 GHz, the radiation boundaries are taken at a distance greater than *λ*_0_/4 from the antenna. The heart muscles are then designed as a cuboid with dimensions of 50 mm × 50 mm × 120 mm, and it is positioned in the center of the radiation box. In order to make the phantom more realistic, skin and fat layers of 4 mm thickness are added on top of the muscle layer, as shown in Fig. 2c. The dielectric properties, such as permittivity and conductivity, of biological tissues (skin, fat, and heart muscle) change with frequency, which are referenced from^30^. The housing of the leadless pacemaker system is designed in the center of the heart muscles (Fig. 2c) The electrode, fixation tines, anode ring, and battery are designated as perfect electric conductors (PECs), while the sensor and other modules are designated as dielectric materials, because the outer layer of a commercial medical device has dielectric properties, whereas the battery has an electrical effect.

### Evolution of the implantable antenna

The MIMO antenna is designed on the Rogers 6010LM substrate (*ε*_*r*_ = 10) with dimensions of 3.4 mm × 6.6 mm × 0.254 mm at the top of the telemetry module of the TCP system. A fractal antenna design is chosen (see Fig. 2d) because fractals increase current paths, which helps in miniaturization and multi-band behavior^31^. The Hilbert curve is one of the most popular fractals, and many researchers have used it to achieve miniaturization in the literature^32–37^. The proposed fractal antenna design is also inspired by the Hilbert curve, and the design procedure of the antenna is as follows:

#### Step-0 (Ant-0)

**Figure 3:**
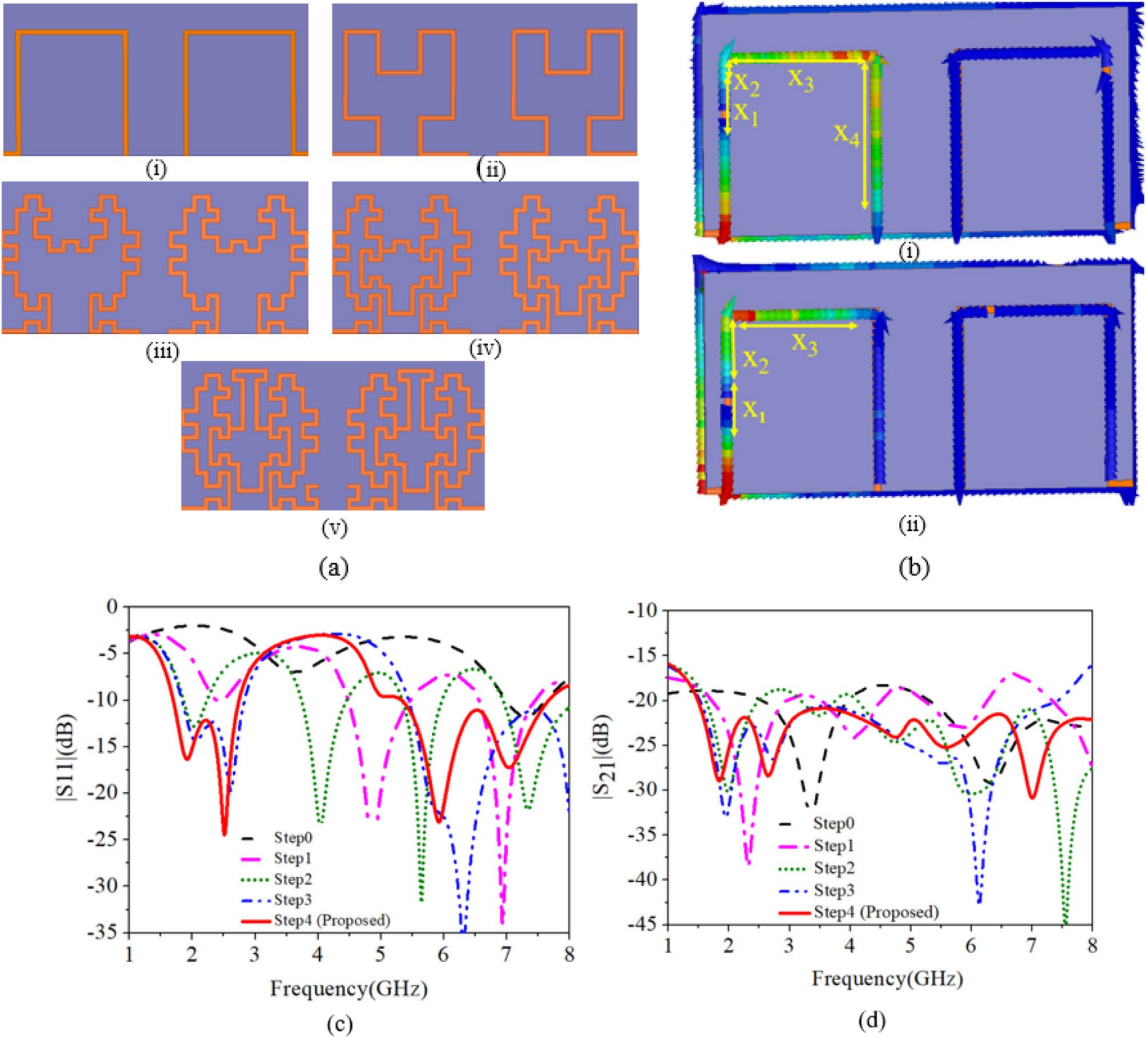
Implantable MIMO antenna (**a**) Evolution steps (i. Step-0, ii. Step-1, iii. Step-2, iv. Step-3, v. Step-4) of the MIMO antenna. (**b**) Current distribution (i. 3.6 GHz, ii. 7.3 GHz) in Step-0. (**c**–**d**) Reflection and transmission coefficients of evolution steps, respectively.

At the targeted frequency of 2.45 GHz, the guided wavelength (*λ*_*g*_) is ~ 17 mm inside the heart muscle, but this much area is not available inside the leadless pacemaker. Therefore, the design begins with an open loop antenna on the substrate of 3.4 mm × 6.6 mm, which is the first stage of the Hilbert curve, as shown in Fig. 3a(i). The Ant-0 resonates in two bands: 3.6 GHz and 7.3 GHz, with poor impedance matching at 3.6 GHz, as shown in Fig. 3b. At 3.6 GHz, the distance between successive current minima (*λ*_*g*_/2) is 5.9 mm, as shown in Fig. 3b(i).

The guided wavelength in the heart muscle is calculated using $\lambda_{g} = \frac{\lambda }{{\sqrt {\varepsilon_{r} } }}$, where $\varepsilon_{r}$ is the permittivity of the heart muscle, which is nearly equal to *λ*_*g*_/2 as shown in the current distribution plot in Fig. 3b(i). Similarly, at 7.3 GHz, the distance between successive current minima is 2.9 mm, as shown in Fig. 3b(ii), which is equivalent to *λ*_*g*_/2, if the permittivity of the heart muscle is considered. Spatial diversity is used to achieve isolation between the antenna radiators by connecting the microstrip feed along the length at both ends, as shown in Fig. 3a. The confinement of the current to the loop (Fig. 3b) also results in good isolation between the two antenna elements because it restricts the interaction of their fields.

#### Step-1 (Ant-1)

The second stage of the Hilbert curve is used (see Fig. 3a(ii)) to shift the resonances to the lower side (2.4 GHz and 5.8 GHz) and improve the impedance matching. As shown in Fig. 3c(i), the antenna resonates in three bands: 2.43 GHz, 4.86 GHz, and 6.93 GHz. The current path length at 2.43 GHz is *λ*_*g*_/2 with poor impedance matching and reflection coefficient of − 6 dB.

#### Step-2 (Ant-2)

The fractal concept is used with little variation in this step. The vertical metal strips of the antenna are bent twice at the center to obtain the 5.8 GHz band, as shown in Fig. 3a(iii), which increases the current path and shifts the lower resonance to 2.1 GHz, 4.86–4.1 GHz, and 6.93–5.65 GHz, and one more resonance appears at 7.3 GHz. Also, 20 dB isolation between the antenna elements is maintained. In this step, good impedance matching, bandwidth, and resonance are obtained in the 5.8 GHz band, but the targeted 2.45 GHz band is shifted to the lower side.

#### Step-3 (Ant-3)

In this step, changes are made to the antenna geometry to merge the 4 GHz band with 2.45 GHz and 7.1 GHz with 5.7 GHz by analyzing the current behavior. The two halves of the antenna elements are connected by a meandered/π-shaped strip, as shown in Fig. 3a(iv). The current flows from one half of the antenna to the other, shifting resonance at 4.86–2.6 GHz and merging with the 1.92 GHz frequency band, resulting in a wide impedance bandwidth of 1.83–2.83 GHz. Also, the 7 GHz band shifts to 6.3 GHz and merges with 5.7 GHz, resulting in a wideband (5.45–8 GHz) at the center frequency of 6.3 GHz. As shown in Fig. 3d(ii), isolation in the higher band at 8 GHz is ~ 15.7 dB, which needs to be improved.

#### Step-4 (proposed antenna (Ant-4))

The main tasks in this step are to improve the isolation in the higher frequency band and shift the 6.3 GHz resonance to 5.8 GHz.

#### Isolation improvement in the higher band

In step-4, a π-shaped metal strip connects the two halves of the antenna elements, as shown in Fig. 3a(v). The total length of this section is 1.2 mm, and by modifying it into a T-shaped stub of length 4.4 mm (0.9*λ*_*g*_ at 8 GHz), a good isolation of > 22.5 dB is achieved.

#### Tuning of 6.3 GHz band to 5.8 GHz band

An open-loop square section is introduced at the end of each antenna element, as shown in Fig. 3a(v), which increases the current path and shifts the 6.3 GHz band to 5.8 GHz. As a result, the proposed antenna covers the ISM 2.45 GHz and 5.8 GHz frequency bands with 1.1 GHz (1.7–2.8 GHz) and 2.33 GHz (5.33–7.6 GHz) bandwidths and isolation of > 22.5 dB in the desired bands, respectively, as shown in Fig. 3c,d.

### Design of outside-body antennas

**Figure 4:**
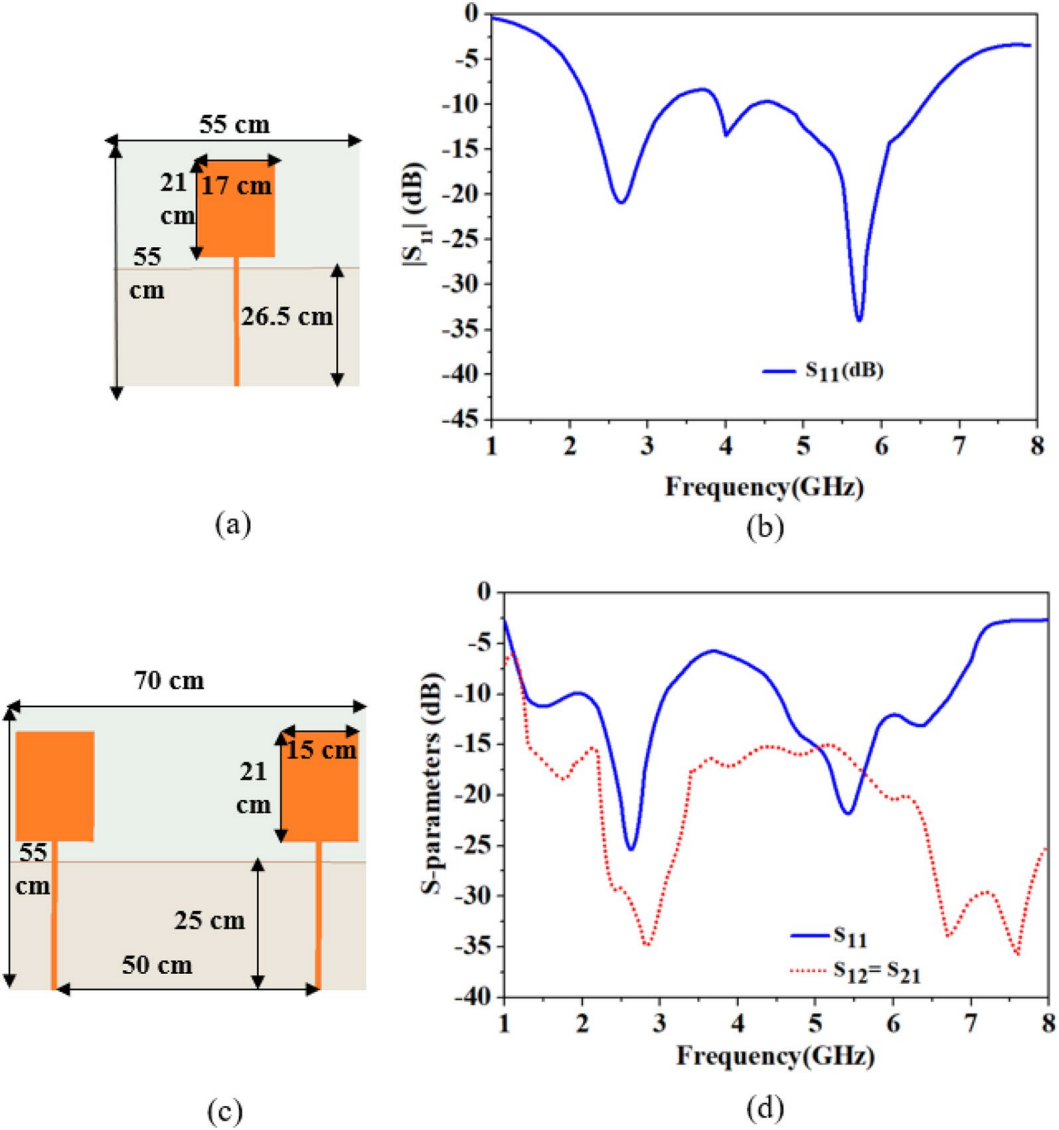
Outside-body antennas (**a**) Single-port patch antenna. (**b**) Reflection coefficients. (**c**) Two-port MIMO antenna. (**d**) S-parameters (reflection and transmission coefficients).

A conventional rectangular patch antenna and a two-port MIMO antenna are designed as outside-body antennas, and fabricated on the Rogers 3003 substrate (*ε*_*r*_ = 3) with a thickness of 0.762 mm. The design of both antennas and their scattering parameters are shown in Fig. 4a–d. In both the designs, the antenna has a partial ground plane and is excited by a microstrip line feed. The partial ground plane not only reduces the size of the antenna but also splits the current vectors and helps in achieving multiple frequency bands. The increased feed length shifts frequencies to the lower side but reduces bandwidth, while ground plane length reduction shifts resonances to the lower side. In the MIMO antenna, two identical antenna elements are positioned in a mirrored-image fashion and spaced 50 cm (4*λ*_0–2.45 GHz_) apart. The reflection coefficients of a single-port antenna are shown in Fig. 4b. Figure 4d shows the reflection and transmission coefficients of the MIMO antenna, where more than 17.5 dB isolation is achieved between antenna elements.

## Results and discussion

### Fabricated prototypes and characterization of human heart muscle phantom

**Figure 5:**
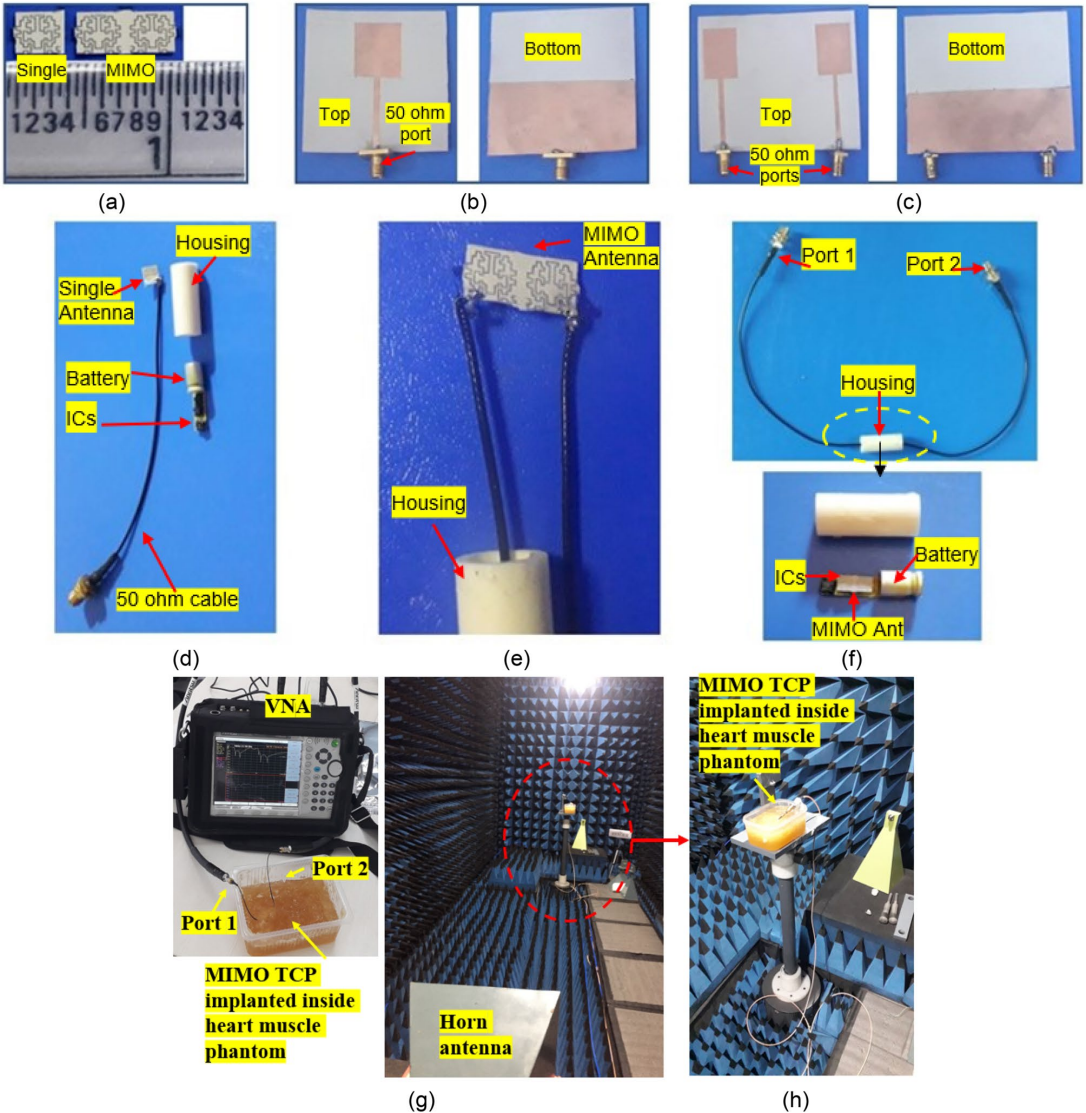
Fabricated prototypes and measurement set-ups. (**a**) Fabricated prototypes of single and two-port MIMO implantable antennas. (**b**) Single-port outside body antenna. (**c**) Two-port outside body MIMO antenna. (**d**) Single-port TCP capsule with antenna and electronics. (**e**) Single-port TCP capsule with antenna and electronics. (**f**) Two-port MIMO antenna with TCP housing. (**g**) Near-field measurement of implantable MIMO antenna. (**h**) far-field measurement of implantable MIMO antenna.

The proposed implantable single-port and two-port MIMO fractal antenna prototypes, and outside-body single-port and two-port MIMO antenna prototypes, are fabricated, and their photographs are shown in Fig. 5a–c. Next, the gel phantom that mimics the properties of heart muscle is prepared. The phantom gel is made with gelatin powder and water, and the process can be found in^38^. The permittivity of the ballistic gel is measured using an open-ended coaxial probe method, which is the most used method for determining the dielectric properties of phantoms. The open-ended coaxial method assumes homogeneous consistency of the phantom. So, air bubbles trapped in the gel may lead to slight differences, which can be reduced by continuously mixing gelatin powder into water and slowly adding it. In this work, the dielectric properties of the ballistic gel phantom are considered for the 2.45 GHz frequency.

### Reflection coefficients and isolation measurement

Figure 5e depicts a fabricated two-port MIMO antenna with copper coated with tin to prevent corrosion. The 50 Ω miniature coaxial cables are connected at both ports of the two-port MIMO antenna. Finally, the fabricated prototype of the MIMO antenna was put inside the ceramic alumina capsule along with IC and battery and sealed at both ends, as shown in Fig. 5f. The *in-vitro* measurement of reflection coefficients and isolation of complete MIMO antenna system in the heart muscle phantom (ballistic gel phantom)^38^ is shown in Fig. 5g.

**Figure 6:**
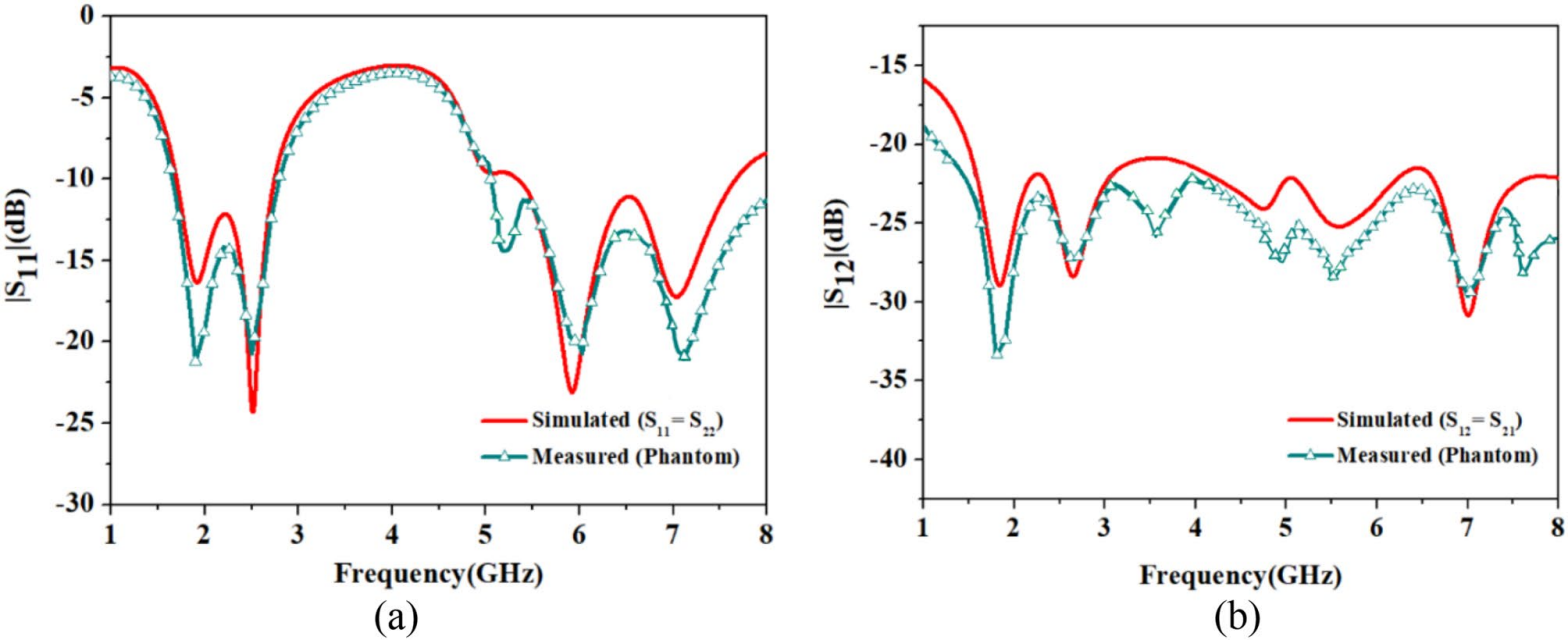
Comparison of the simulated and measured S-parameters plot of the proposed TCP MIMO antenna system: (**a**) |S_11_| =|S_22_|. (**b**) |S_12_| =|S_21_|.

**Figure 7:**
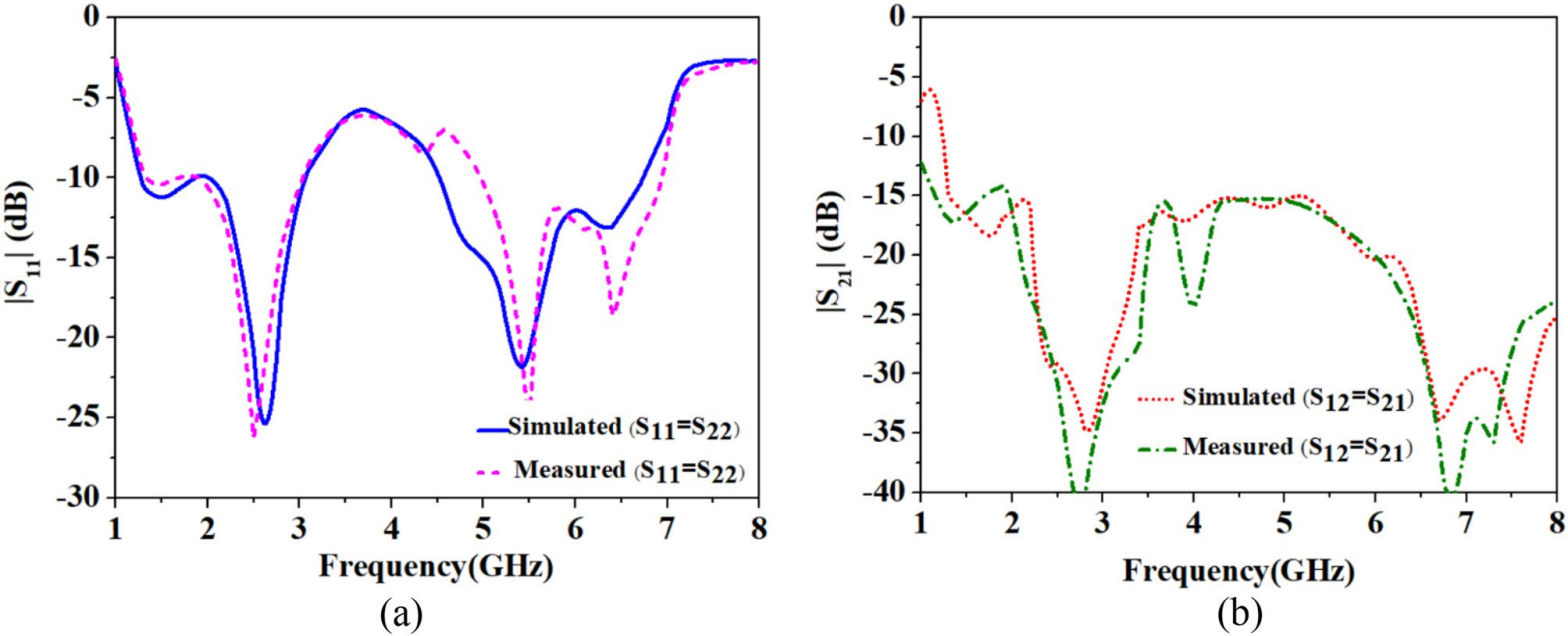
Comparison of the simulated and measured S-parameters outside body MIMO antenna system: (**a**) |S_11_| =|S_22_|. (**b**) |S_12_| =|S_21_|.

Next, comparison of the measured and simulated reflection coefficients (|S_11_| and |S_22_|) of Antenna 1 and Antenna 2 and isolation (|S_12_| and |S_21_|) between antenna elements is shown in Fig. 6. While measuring the antenna’s performance in the heart muscle’s gel phantom, multiple resonances show up, which may possibly be due to air bubbles in the phantom, length of the coaxial cable, and implantation depth of the capsule in the phantom. Irrespective of the variations, the measured results were as expected. Impedance bandwidths of 1.2 GHz and 3 GHz are achieved in the heart muscle gel phantom at the resonant frequency of 2.45 GHz and 5.8 GHz, respectively. The isolation between the antenna elements is also good, and its measured value is 23.2 dB, which is close to the simulated value of 22.6 dB. Next, the S-parameters of outside-body two-port MIMO (Fig. 5c) antenna in the air are shown, using vector network analyzer. Figure 7a,b show measured value of reflection coefficients and transmission coefficients (isolation) and their comparison with the simulated results (achieved in Ansys HFSS).

**Figure 8:**
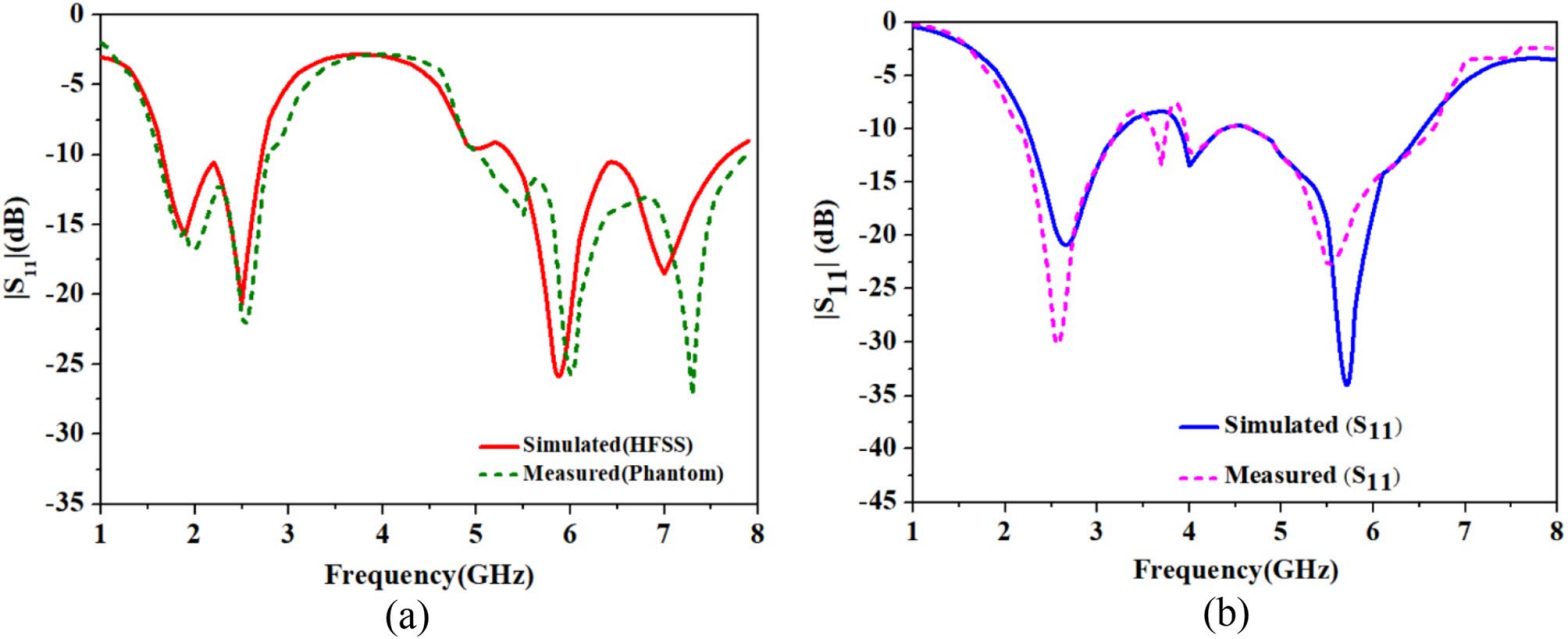
Comparison of the simulated and measured S-parameters: (**a**) single port implantable antenna, (**b**) single port outside body antenna.

At 2.45 GHz, the simulated and measured impedance bandwidths are 1.26 GHz (1.74–3.00 GHz) and 1.22 GHz (1.73–2.95 GHz), respectively, and, at 5.8 GHz, the simulated and measured impedance bandwidths are 1.7 GHz (4.5–6.2 GHz) and 1.91 (5.00–6.91 GHz), respectively. As shown in Fig. 7b, simulated and measured values of isolation in the ISM 2.45 GHz is 17.5 dB and 18.3 dB, respectively, and, at 5.8 GHz, simulated and measured isolation are 18.4 dB and 18.3 dB, respectively. Hence, the measured S-parameters are in good agreement with the simulated parameters, in both cases: implantable and outside body MIMO antennas (Fig. 8).

Single-port implantable antenna and outside-body antennas fabricated prototypes are shown in Fig. 5a,b, respectively. Single-port implantable antenna is also integrated into another ceramic alumina capsule along with ICs and battery, as shown in Fig. 5d, and reflection coefficients are measured into the heart muscle phantom using vector network analyzer. The simulated and measured impedance bandwidths at the 2.45 GHz are 1.05 GHz (2.25–3.3 GHz) and 1.0 GHz (3.2–2.2 GHz), respectively, and, at 5.8 GHz, simulated and measured impedance bandwidths are 1.8 GHz (4.7–6.5 GHz) and 1.9 GHz (4.7–6.6 GHz), respectively.

### Radiation pattern measurement

**Figure 9:**
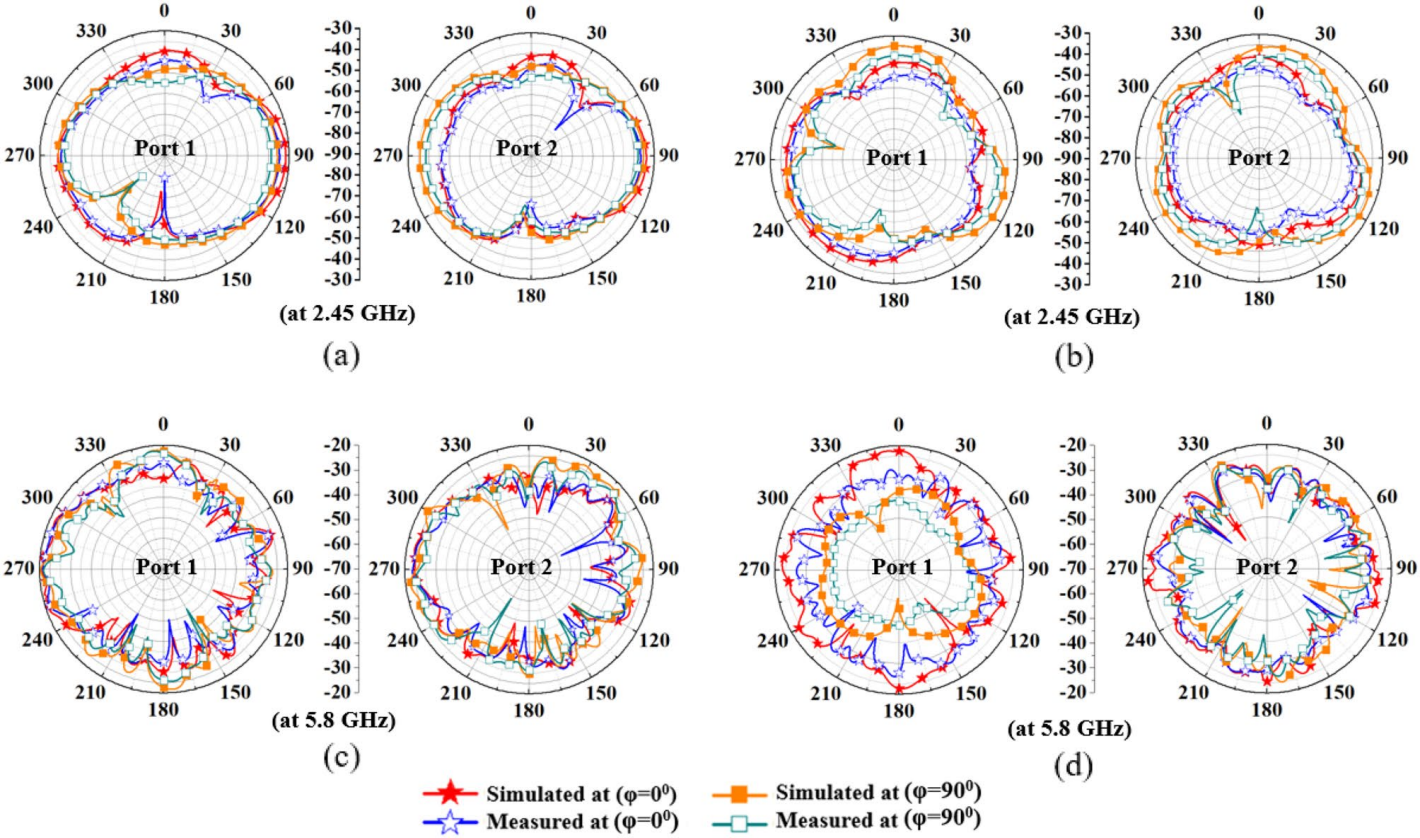
Comparison of simulated and measured radiation patterns of the proposed antenna (**a**) H-plane (at 2.45 GHz) (**b**) E-plane (at 2.45 GHz) (**c**) H-plane (at 5.8 GHz) (**d**) E-plane (at 5.8 GHz).

The gain of the MIMO antenna in the muscle phantom is measured in an anechoic chamber, as shown in Fig. 5h. The simulated and measured peak gains are demonstrated in Table 1 (boresight direction (*θ* = 0˚)). The measured gain values are less than the simulated values, likely due to implantation depth variation (because of human error) in the muscle phantom during measurements. It is very common for ultra-compact implantable antennas to have low gain values due to high losses in biological tissues, as per^39^. Therefore, the proposed antenna has the minimum gain value when compared to the other reported implantable MIMO antennas in Table 2. According to Fig. 9, radiation patterns of Antenna 1 and Antenna 2 in both the *E* (*ϕ* = 0˚) and *H* (*ϕ* = 90˚) planes demonstrate diversity, making the proposed antenna in an integrated approach an attractive entrant for efficient wireless communication in a lossy human body environment.

**Table 1:** Peak gain (dBi) of the antennas.

Frequency (GHz)	Implantable single antenna: simulated/measured	Implantable MIMO antenna: simulated/measured	Outside-body single antenna: simulated/measured	Outside-body MIMO antenna: simulated/measured
2.45	− 30.4/− 31.6	− 31.6/− 33.2	2.8/2.7	3.79/3.74
5.8	− 22.2/− 23.5	− 21.4/− 24.3	3.6/3.45	4.87/4.8

**Table 2:** Comparison of the proposed antenna with other reported antennas.

References	^14^	^15^	^16^	^17^	^18^	^29^	Prop
Antenna type	Loop/dipole	Spiral	Loop (circular/rectang.)	EBG	Circular patch	Meandered	Fractal loop
Profile	Conformal	Flat	Conformal	Flat	Flat	–	Flat
Number of elements	2	2	2	4	2	4	2
Isolation (dB)	12	25	18	16	26	20	22.6
Area ($\lambda_{g}$^2^)	1.1 $\lambda_{g}$^2^/0.9 $\lambda_{g}$^2^	4.4 $\lambda_{g}$^2^	6.5 $\lambda_{g}$^2^/6.6 $\lambda_{g}$^2^	1.17 $\lambda_{g}$^2^	0.98 $\lambda_{g}$^2^	0.109 $\lambda_{g}$^2^	0.072 $\lambda_{g}$^2^
Area (mm^2^)	113/91	484	78.5/113.6	342.2	100.2	32.24	22.4
Freq. (GH_z_)	0.433	0.402	2.45	2.45	403/433	2.45	2.4/5.8
Ground plane	No	Partial	No	Slotted	Slotted	Slotted	Full
Implant depth (mm)	100	4	30	19.5	4	50	60
BW (%)	2.7	35.9	16	18.64	33.9	14.2	44/40
Peak gain (dBi)	− 25	− 36.8	–	− 15.1	− 30	− 20	− 31.6/− 21.4
SAR (W/kg) 1 g/10 g (at 1W)	–	–	–	–	– –/550	1.58 (at 0.025 W)	263.3/43.2 107.45/17.31
ECC	–	–	–	0.0025	0.1	0.12	0.005/0.0014
Application	Endoscopy capsule	–	Endoscopy capsule	–	Endoscopy capsule	Endoscopy capsule	Leadless pacemaker capsule

**Figure 10:**
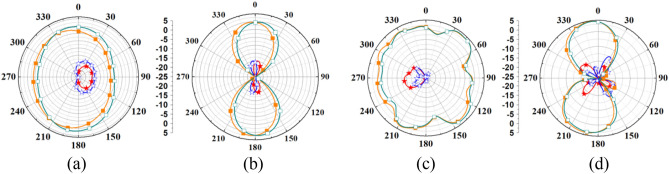
Comparison of simulated and measured radiation patterns of external MIMO antenna (at Port 1) (**a**) H-plane (at 2.45 GHz) (**b**) E-plane (at 2.45 GHz) (**c**) H-plane (at 5.8 GHz) (**d**) E-plane (at 5.8 GHz).

**Figure 11:**
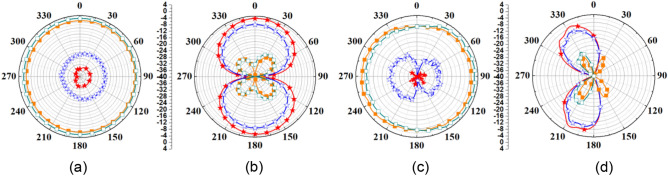
Comparison of simulated and measured radiation patterns of external single-port antenna (**a**) H-plane (at 2.45 GHz) (**b**) E-plane (at 2.45 GHz) (**c**) H-plane (at 5.8 GHz) (**d**) E-plane (at 5.8 GHz).

**Figure 12:**
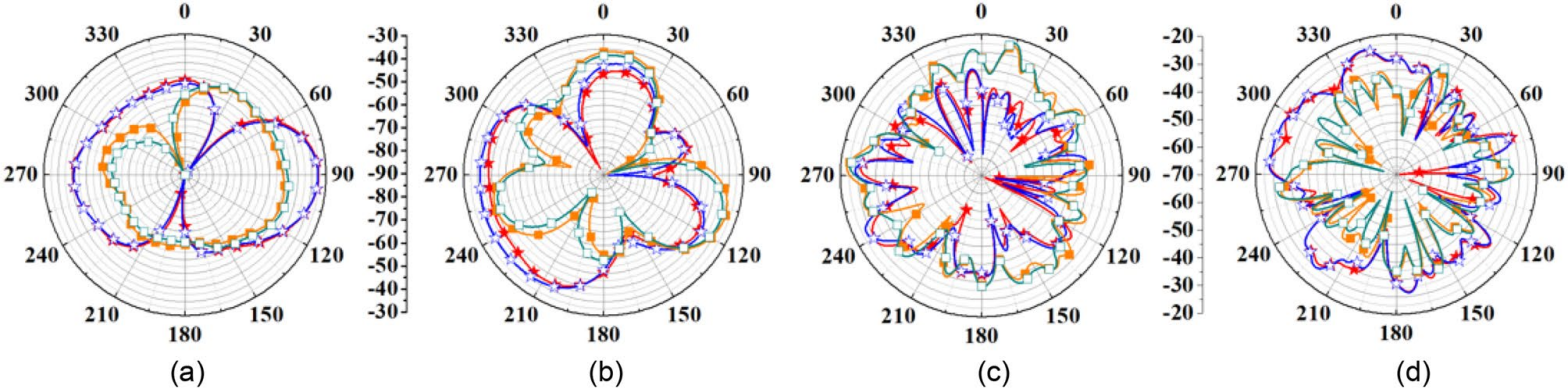
Comparison of simulated and measured radiation patterns of implantable single-port antenna (**a**) H-plane (at 2.45 GHz) (**b**) E-plane (at 2.45 GHz) (**c**) H-plane (at 5.8 GHz) (**d**) E-plane (at 5.8 GHz).

A comparison of simulated and measured radiation patterns of outside-body two-port MIMO antenna at 2.45 GHz and 5.8 GHz is shown in Fig. 10. It can be noticed that the radiation patterns of the antenna are almost omnidirectional in the *H*-plane. At 2.45 GHz, simulated and measured peak gain values are 3.79 dBi and 3.74 dBi, respectively. Radiation patterns for single-port outside-body antenna and single-port implantable antennas are shown in Figs. 11 and 12. In the case of single-port outside-body antenna, radiation pattern is omnidirectional in the *H*-plane at both the resonant frequencies (ISM 2.45 and 5.8 GHz), and in the *E*-plane, radiation pattern is eight-shaped. Simulated and measured values of the peak gains for outside-body single-port antennas are 2.8 dBi and 2.7 dBi, respectively, at 2.45 GHz frequency. Whereas, at 5.8 GHz, simulated and measured peak gains are 3.6 dBi and 3.45 dBi, respectively.

**Figure 13:**
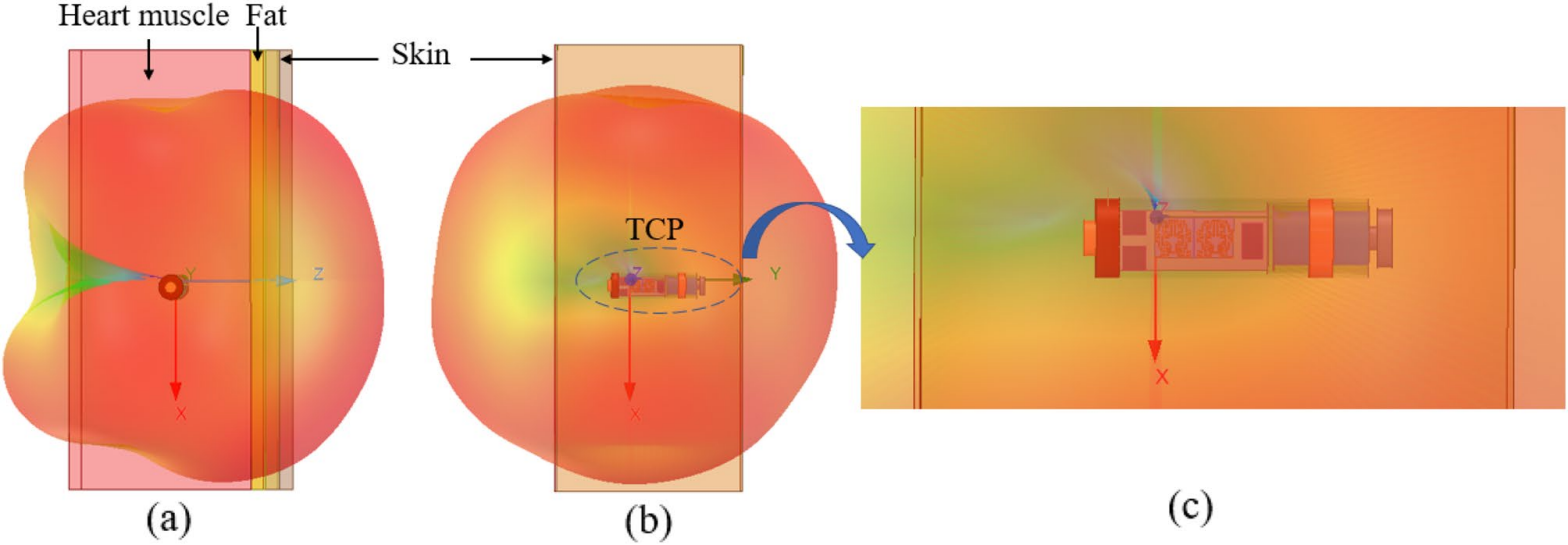
3D Radiation pattern of the MIMO integrated antenna at 2.45 GHz (**a**) side view (**b**) front view (**c**) zoom view of MIMO integrated TCP system along with the required electronics.

**Figure 14:**
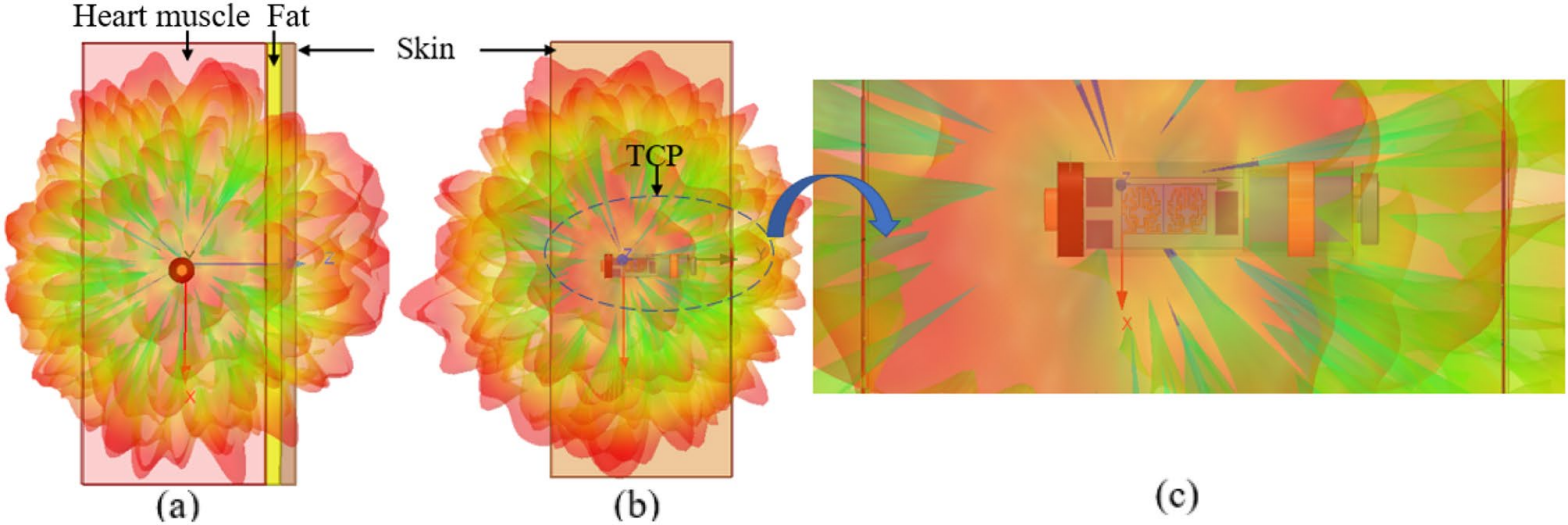
3D Radiation pattern of the MIMO integrated antenna at 5.8 GHz (**a**) side view (**b**) front view (**c**) zoom view of MIMO integrated TCP system along with the required electronics.

Single-port implantable antenna was integrated into the biocompatible ceramic alumina’s capsule housing along with ICs and battery, as shown in Fig. 5d, later, its radiation pattern was measured in an anechoic chamber by placing the integrated TCP system into the heart muscle phantom (in the same way as radiation pattern of the two-port implantable MIMO antenna was measured). The simulated and measured peak gain values at 2.45 GHz are − 30.4 dBi and − 31.6 dBi, respectively, and, at 5.8 GHz, simulated and measured peak gain values are − 22.2 dBi and − 23.5 dBi, respectively. For further clarification, simulated 3D radiation patterns of the MIMO integrated TCP system are shown in Figs. 13a–c and 14a–c at 2.45 and 5.8 GHz, respectively, when both the ports of MIMO antenna are excited. It is clear from Figs. 13 and 14 that the proposed MIMO antenna is capable of radiating outside body efficiently in both the planes (azimuth and elevation) and proves its usefulness for stable communication to the outside body devices.

### Envelope correlation coefficient (ECC) and diversity gain (DG)

The envelope correlation coefficient (ECC) of a MIMO antenna indicates how independent the antenna elements are of one another. Ideally, there should be zero correlation between the antenna elements in order to avoid undesired coupling of the power radiated by each antenna. But practically, ECC < 0.5 is acceptable. ECC is calculated with the help of the 3D radiation pattern, as explained in^40^.

1 $$ ECC = \frac{{\left| {\iint_{4\pi } {\overrightarrow {{F_{i} }} \left( {\theta ,\Phi } \right) \cdot \vec{F}_{j} \left( {\theta ,\Phi } \right)^{*} \partial \Omega }} \right|^{2} }}{{\iint_{4\pi } {\left| {\overrightarrow {{F_{i} }} \left( {\theta ,\Phi } \right)} \right|^{2} } \partial \Omega \cdot \iint_{4\pi } {\left| {\overrightarrow {{F_{j} }} \left( {\theta ,\Phi } \right)} \right|^{2} }\partial \Omega }} $$

**Figure 15:**
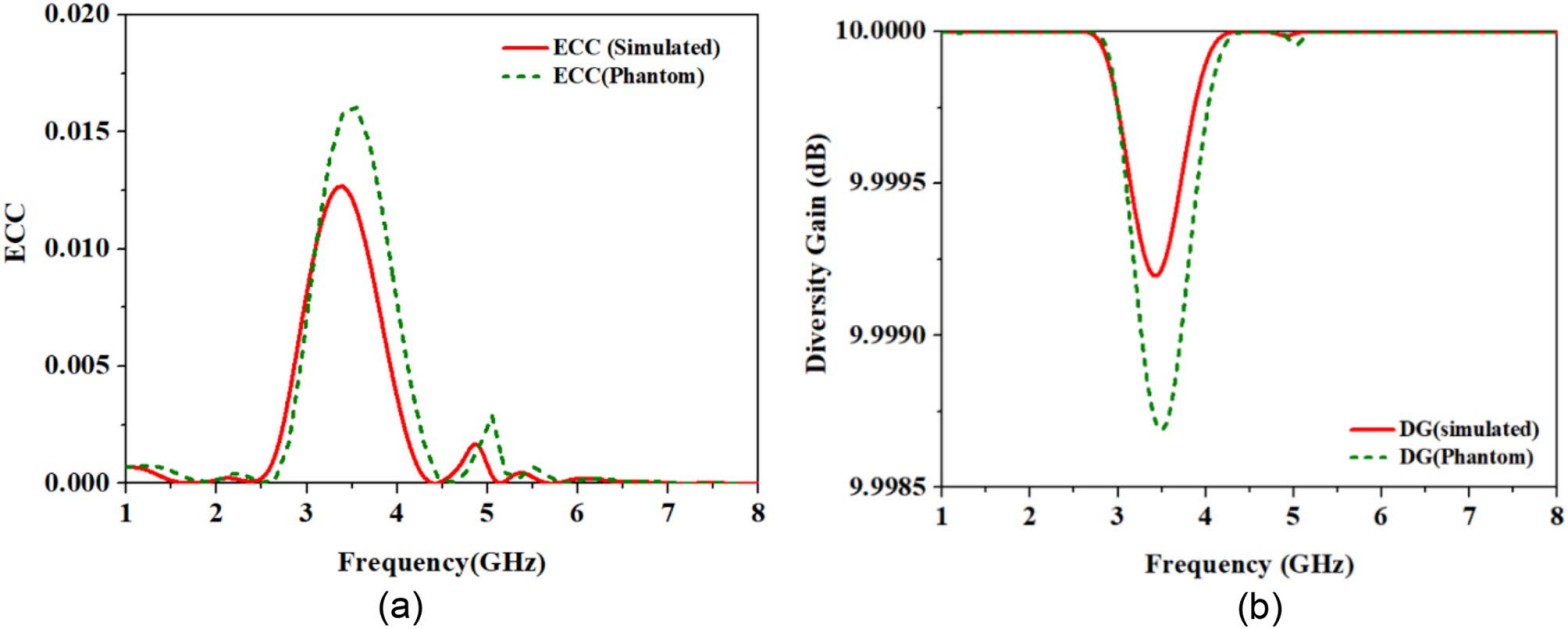
Comparison of simulated and measured parameters of the MIMO antenna TCP system: (**a**) ECC (**b**) DG.

It can be noted from Fig. 15a that the minimum and maximum values of simulated and measured ECC are 0.0012 and 0.0017 and 0.0052 and 0.0045, respectively, in entire first frequency band (2.45 GHz). Whereas in the second band: 5.8 GHz, simulated and measured maximum values of ECC are 0.0014 and 0.0016, which makes this antenna suitable for MIMO communications. Likewise, the diversity gain (DG) is another essential parameter to evaluate the MIMO system performance. The effect on the transmitted power due to the diversity scheme is evaluated by DG. Ideally, it should be 10 dB, indicating zero correlation between the antenna elements. The higher DG indicates less coupling between the channels of the MIMO antenna or a higher signal-to-interference ratio. The DG can be calculated using the following equation^41^.

2 $$ DG = 10\sqrt {1 - ECC^{2} } $$

Simulated and measured DG is close to 10 dB, as shown in Fig. 15b.

### Specific absorption ratio (SAR)

**Figure 16:**
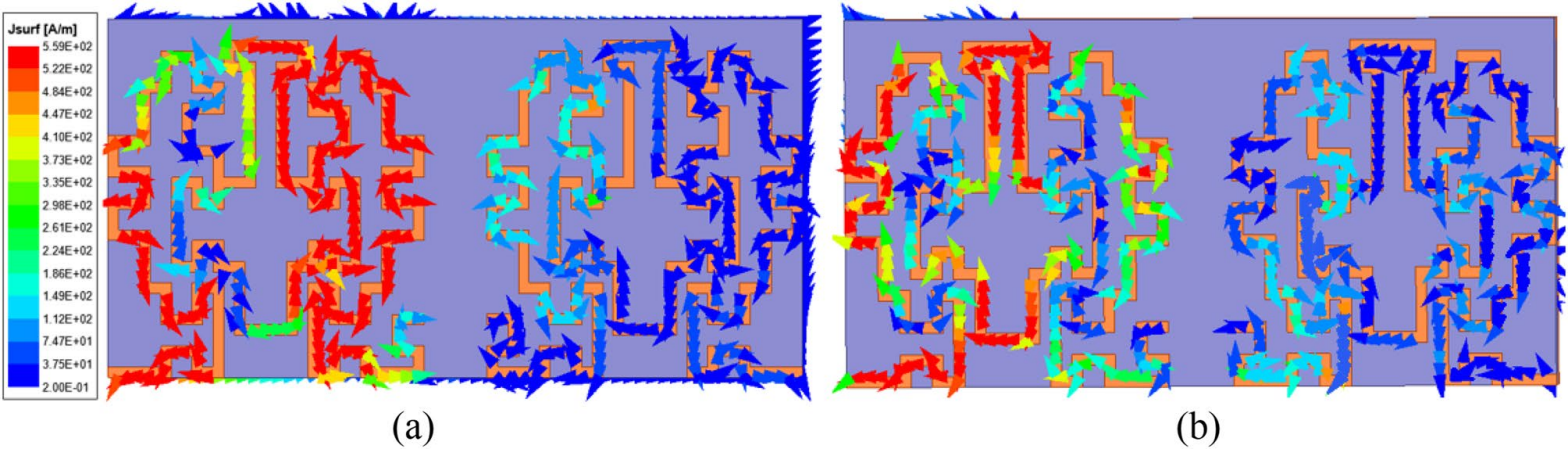
Current distribution (when port 1 is excited): (**a**) at 2.45 GHz (**b**) at 5.8 GHz.

**Figure 17:**
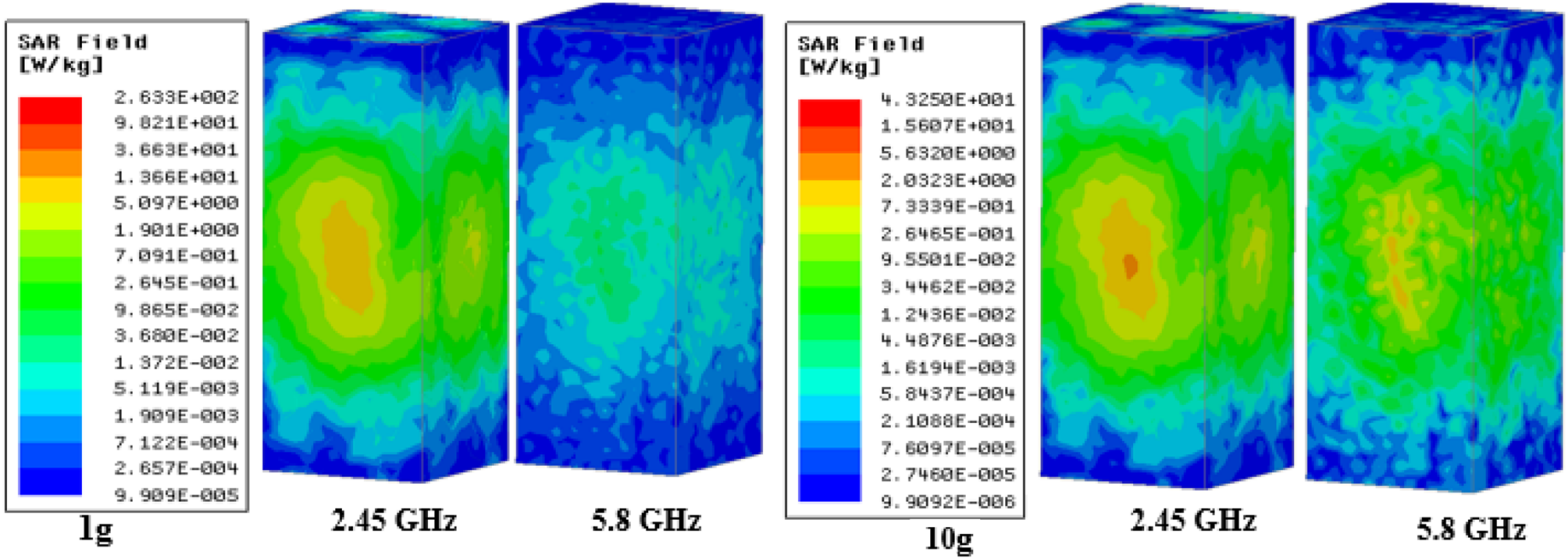
Distribution of SAR in the canonical heart model.

The proposed TCP MIMO antenna system is checked in terms of safety by evaluating the SAR when the prototype lies in the multilayer human heart phantom. For this purpose, the new guidelines of International Commission on Non ionizing Radiation Protection (ICNIRP) and IEEE C95.1-2019 standards^42^ are considered. As per the safety standards the Average SAR (ASAR) value for 1 g/10 g of human tissue in a shape of a cube cannot be greater than 1.6/2.0 W/kg. All the SAR values (1 g/10 g) correspond to the input power of 1 W. Although ASAR value should be high at high frequency, in the present case, it is low because at 5.8 GHz intensity of the current is low as compared to the 2.45 GHz and causes a low value (1 g/10 g ASAR values are 107.45/17.31) of SAR at high frequency, as shown in Fig. 16. Whereas for low resonance (2.45 GHz), a comparatively high-intensity current was observed and led to a higher 1 g/10 g ASAR value of 263.3/43.2 W/Kg, the corresponding maximum allowable input power is 6.07/ 46.2 mW, respectively, to meet the standard limit, as shown in Fig. 17. The power requirement of a leadless pacemaker is only 5–10 µW, as per^43^; hence, on the basis of results, the MIMO TCP system will be safe for long-term use inside the human body.

### Far-field measurement set-up for SISO communication and MIMO communication in the simulator and anechoic chamber

In this section, the single-port antenna TCP communication system and MIMO antenna TCP communication system in the simulator and anechoic chamber (for experimental verification) are explained and compared. In^12^, a MIMO system was used to extend the transmission range for non-biomedical applications. The same concept is tested in this work for implantable TCP system as it is also enabled with remote monitoring, and by using the MIMO communication technology, one can achieve increased transmission range (or received power for fixed distance), hence improving the performance of the TCP system. Furthermore, with the deployment of 5G networks, there is a strong need to replace conventional single port antennas with MIMO antennas, as MIMO antennas are one of the requirements for 5G communication. Considering all of these factors, SISO and MIMO communications are established and compared in the simulation and anechoic chamber to validate the advantage of MIMO communication over SISO communication.

#### Single-port antenna TCP communication system (simulated and measured)

**Figure 18:**
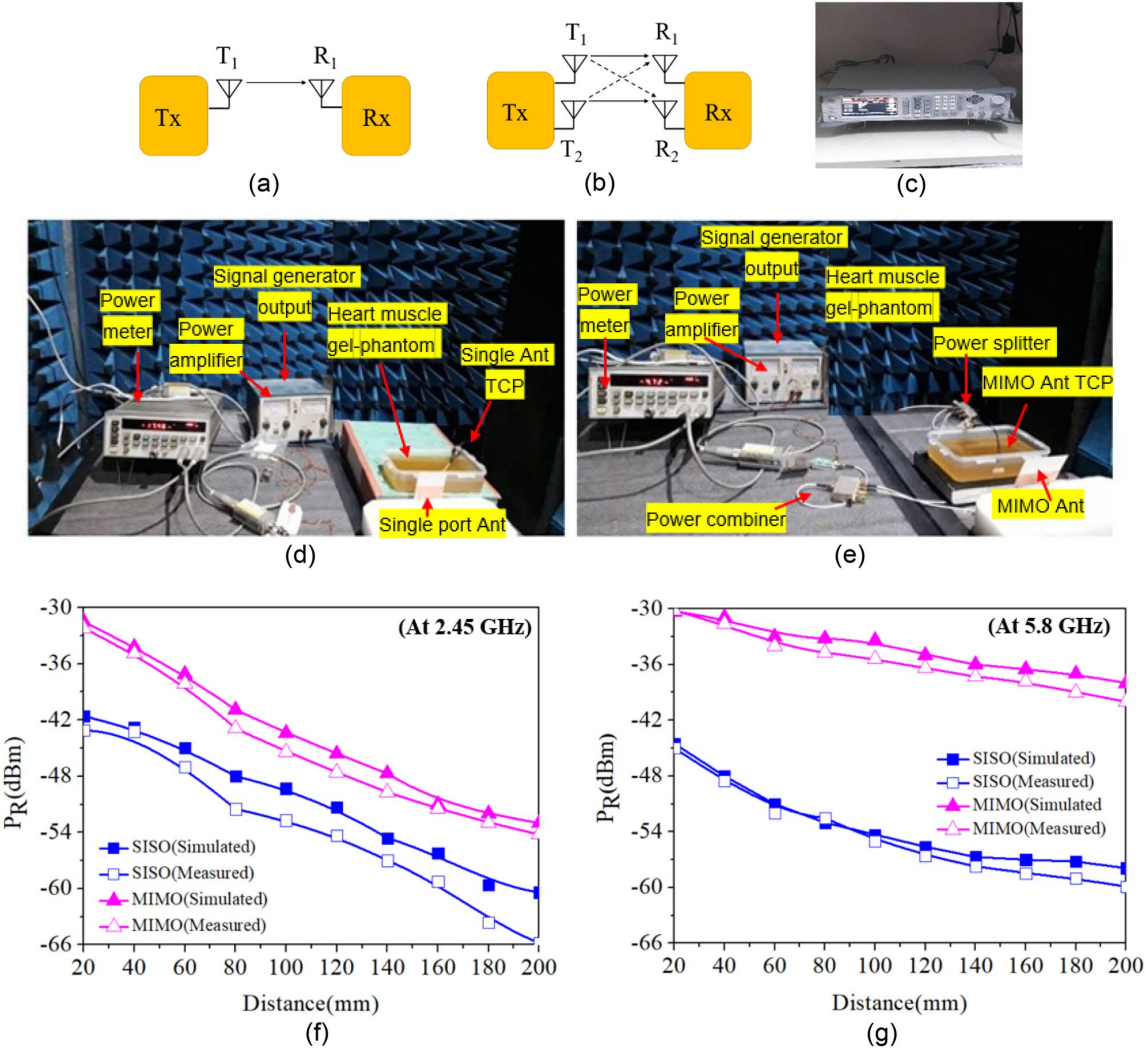
(**a**) Block diagram of SISO communication. (**b**) Block diagram of MIMO communication (**c**) signal generator (**d**) and (**e**) measurement set-up for SISO and MIMO communication systems. (**f**) Comparison of simulated and measured received power at 2.45 GHz for SISO and MIMO communications. (**g**) Comparison of simulated and measured received power at 5.8 GHz for SISO and MIMO communications.

In a leadless pacemaker communication system, an external device receives data wirelessly from the implanted device (TCP) and transmits it wirelessly to a healthcare professional. The implanted leadless pacemaker (transmitter) and the external device (receiver) that receives the patient's data use a single-port antenna for both transmission and reception, which is referred to as a SISO communication system, as shown in Fig. 18a. This type of communication is currently used in pacemakers like Medtronic's micra TCP system.

##### Implementation in the simulating environment:

A single antenna pacemaker consists of a dual-band (ISM 2.45 GHz/5.8 GHz) antenna, battery, and ICs that are integrated inside a biocompatible ceramic alumina capsule housing, as shown in Fig. 18d. The TCP capsule with integrated antenna and electronics is implanted into the heart muscle phantom in the simulator HFSS, and the external dual-band antenna (shown in Fig. 18d) is placed at the far-field distance. At both the ISM 2.45 and 5.8 GHz bands, the far-field distance for the high permittivity lossy heart muscle is greater than (2*D*^2^/*λ*_*g*_), where *D* is the largest dimension of the antenna and $\lambda_{g} = \frac{\lambda }{{\sqrt {\varepsilon_{r} } }}$, and $\varepsilon_{r}$ is the permittivity of the heart at the resonating frequency.

In the present case, the far-field distance is 5.1 mm at 2.45 GHz and 12.13 mm at 5.8 GHz. Thus, distances greater than 12 mm are in the far-field range. In this work, a distance of 20–200 mm is used to compare the communication system in terms of losses or received power strength. Therefore, for the evaluation of the received power strength, distances ranging from 20 to 200 mm are taken into account, and the received power is calculated using the transmission coefficient (*S*_21_) between the implantable TCP antenna (transmitter) and the single-port antenna (receiver) using the following formula:

3 $$ \left| {S_{21} } \right|^{2} = \frac{{P_{R} }}{{P_{T} }} $$

The amount of transmitted power is considered as per the maximum SAR values that the human body can tolerate without damaging biological tissues. SAR values are evaluated to validate the safety of the proposed communication system in accordance with the Federal Communications Commission (FCC) and ICNIRP guidelines^44^. Figure 17 shows 1 g/10 g SAR values at 2.45 GHz and 5.8 GHz. The maximum allowable power to meet the standard limit is 6.07/46.2 mW, respectively for the highest 1 g/10 g ASAR value of 263.3/43.2 W/Kg at 2.45 GHz. At 5.8 GHz, 1 g/10 g ASAR values are 107.45/17.31, resulting in a maximum allowable power of 14.8/155 mW, respectively. In order to ensure the patient's safety, the maximum input power is set to 8 dBm (6.3 mW) at 2.45 GHz and 11 dBm (12.5 mW) at 5.8 GHz.

Figure 18f depicts the received power strength at the outside-body antenna in the simulator for SISO system. At 2.45 GHz, the maximum received power (*P*_*R*_) at 20 mm distance (minimum) is − 42.4 dBm, while at 200 mm distance, the power received is − 58 dBm. Whereas, at 5.8 GHz, the maximum received power is − 44.2 dBm at 20 mm and − 57 dBm at 200 mm, as shown in Fig. 18g.

##### Implementation in anechoic chamber

The received power measurements for single-port antenna communication are performed inside the anechoic chamber, as shown in Fig. 18d. The RIGOL RF signal generator, which feeds transmitting TCP antenna, is located outside the anechoic chamber, as shown in Fig. 16c.

The TCP system is immersed in the heart muscle gel phantom and fed by 8 dBm power (at 2.45 GHz) and 11 dBm power (at 5.8 GHz) from the signal generator via a power amplifier, as shown in Fig. 18d. The external dual-band antenna is placed outside the phantom container to receive power from the TCP system. The power received by the external antenna is measured using a power meter, as shown in Fig. 18d. The received power is measured through the external antenna and plotted in Fig. 18f,g at various distances (20–200 mm) from the implantable TCP system. The measured value of received power at 20 mm distance is − 43.5 dBm and − 45.2 dBm at 2.45 GHz and 5.8 GHz, respectively, whereas at 200 mm distance, received power is − 66 dBm at 2.45 GHz and − 60 dBm at 5.8 GHz.

#### MIMO antenna TCP communication system (simulated and measured)

Multiple antennas are used on the transmitter and receiver sides of the MIMO communication system. The proposed work has considered a (2 × 2) MIMO antenna system, as shown in Fig. 18b. The total power received at the receiving end is given by,

4 $$ P_{R} = P_{R11} + P_{R21} + P_{R22} + P_{R12} $$

where $P_{R11}$ is received power at the receiving antenna *R*_1_ due to transmitting antenna *T*_1_, $P_{R21}$ is the received power at the receiving antenna *R*_1_ due to transmitting antenna *T*_2_, $P_{R22}$ is the received power at the receiving antenna *R*_2_ due to transmitting antenna *T*_2_, and $P_{R12}$ is the received power at the receiving antenna *R*_2_ due to transmitting antenna *T*_1_. In this case, a two-port dual-band MIMO TCP antenna is used on the transmitter side, and a two-port dual-band patch antenna is used on the receiver side.

All received powers ($P_{R11} ,P_{R21} ,P_{R22} ,{\text{ and}} P_{R12}$) are calculated from the transmission coefficients between the transmitting and receiving antennas for 8 dBm and 11 dBm of transmitted power at 2.45 GHz and 5.8 GHz, respectively. Therefore, the total received power *P*_*R*_ can be calculated by using Eq. (2). At 20 mm distance, the received power is − 31 dBm at 2.45 GHz and − 30 dBm at 5.8 GHz. At 200 mm distance, the received power is − 52 dBm and − 37.5 dBm at 2.45 GHz and 5.8 GHz, respectively.

##### Implementation in anechoic chamber

The communication set-up is also established in the anechoic chamber. The proposed TCP MIMO antenna system is immersed in the ballistic gel heart muscle phantom. The output of the signal generator is fed into the power amplifier, which is then fed to the 3-dB power splitter. The power splitter supplies equal power to both antenna elements. The power transmitted by the implantable MIMO antenna TCP system is received by the external MIMO antenna and measured through a power meter, as shown in Fig. 16e. In this case, the minimum received power is − 52.8 dBm at 2.45 GHz and − 38 dBm at 5.8 GHz at 200 mm distance. The simulated and measured results are in good agreement, as shown in Fig. 18f,g.

When SISO and MIMO antenna communication schemes are compared in terms of received power strength, the SISO communication system receives the least amount of power at both (2.45 GHz and 5.8 GHz) resonances, as shown in Fig. 18f,g. In contrast, the received power in MIMO communication is much higher. However, the minimum received power and communication range in MIMO communication are greater at 5.8 GHz than at 2.45 GHz because the gain of the transmitting and receiving MIMO antennas is greater in the 5.8 GHz band than in the 2.45 GHz band. Hence, it can be concluded that MIMO communication achieves maximum received power when compared to SISO communication.

## Limitations and future scope

The presented work uses a single port monopole and two-port MIMO monopole antennas as outside-body antennas to demonstrate single port/MIMO antenna communications. The omnidirectional radiation pattern of the monopole antenna makes it a good choice for outside-body communication as there is no need for alignment with the implantable antenna. Furthermore, by using a high gain omni-directional MIMO antenna array instead of a simple monopole antenna increases the communication range or reduces the losses for fixed communication distance. Also, this study found that MIMO antenna communication not only provides long range/low-loss communication for present IMDs, but it is also compatible with future IMDs on the 5G-IoT platform and can participate in real-time monitoring of human-heart for future leadless pacemakers.

## Conclusion

This paper proposes an ultra-compact MIMO antenna integrated into the leadless pacing system, which enables low-loss MIMO communication to the external MIMO antenna for remote cardiac monitoring on 5G IoT platform. Here, in order to check the low-loss capability of MIMO communication, two types of far-field communication systems are realized: one with a single-port antenna and the other with MIMO antenna in the simulator and anechoic chamber. The performance of both communication systems is compared by comparing their received power. A comparison of simulated and experimental results shows that MIMO antenna communication system receives more power than SISO communication system at both frequency bands (ISM 2.45 GHz/5.8 GHz). Hence, due to its ultra-compact size and higher far-field received power in both frequency bands, the proposed MIMO antenna communication system is suitable for remote health monitoring and wireless power transfer for commercial leadless TCP systems: Micra's TCP and Nanostim's LCP in 4G as well as in 5G IOT based remote monitoring applications.

## Data Availability

The datasets used and/or analyzed during the current study are available from the corresponding author on reasonable request.
